# Identification of Quality Indicators Used to Monitor, Evaluate and Improve Rural and Remote Care for Older People: A Scoping Review

**DOI:** 10.1111/ajr.70105

**Published:** 2025-11-10

**Authors:** Jenni Suen, Rangika L. Fernando, Maria C. Inacio, Maria Crotty, Xiaoping Lin, Gillian E. Caughey

**Affiliations:** ^1^ College of Medicine and Public Health, Flinders Health and Medical Research Institute, Rehabilitation, Aged and Extended Care Flinders University Adelaide Australia; ^2^ Registry of Senior Australians Research Centre South Australian Health and Medical Research Institute Adelaide Australia; ^3^ Registry of Senior Australia Research Centre, College of Nursing and Health Sciences, Caring Futures Institute Flinders University Adelaide Australia; ^4^ Centre for Healthy Brain Ageing, Discipline of Psychiatry & Mental Health, School of Clinical Medicine The University of New South Wales Kensington New South Wales Australia; ^5^ Clinical Outcomes Data Reporting and Research Program, School of Public Health and Preventive Medicine Monash University Melbourne Victoria Australia

**Keywords:** geriatric, health care quality indicator, quality benchmarking, quality measure

## Abstract

**Objective:**

Quality indicators (QIs) specific to older adults receiving health care in rural and remote settings can be used to monitor healthcare quality, inform service improvements, and outcomes for these populations. This scoping review aimed to identify population‐based QIs used to evaluate healthcare quality received by older people in rural and remote settings.

**Methods:**

Two academic databases and grey literature sources were searched to identify population‐based rural QI monitoring programs or rural QIs, routinely used and reported since 2012. QI program and specific characteristics, including country of origin, dimension of care quality captured, domain represented, QI type, data sources used, reporting strategies, and care settings were summarised.

**Findings:**

Nine QI programs from seven countries with 52 QIs were identified. The QIs measured quality‐of‐service delivery (*n* = 28, 54%), accessibility (*n* = 11, 21%), resources (*n* = 9, 17%), and hospital readmissions (*n* = 4, 8%). Most QIs were outcome (*n* = 25, 48%) or process (*n* = 19, 37%) indicators, considering the dimension of safe healthcare. Three QIs (6%) measured rural hospital readmission in older people residing in rural areas.

**Conclusions:**

Three QIs measuring the proportion of older adults with unplanned rural hospital readmission were identified that could facilitate consistent reporting and benchmarking of care provided to older adults residing in rural and remote communities. Given the known disparities in equitable access and quality of healthcare for older people residing in rural and remote areas, these findings highlight the need for QIs across all quality dimensions to monitor healthcare quality and drive improvements in access and quality of healthcare.

**Summary**

- What is already known on this subject?
  ○ One third of older Australians live in rural and remote settings.
  ○ Australia is amongst 13 countries that have national quality monitoring programs, measuring quality indicators on the care provided to older adults in long‐term care.
  ○ Quality indicators are standardised, evidence‐based measures that can be used to facilitate and advocate for appropriate resource allocation in the hospital, community and long‐term care settings.
  ○ American quality monitoring programs have identified that quality indicators used need to be specific to rural settings to ensure the healthcare needs in rural and remote settings are appropriately represented.
- What this paper adds?
  ○ This paper provides a comprehensive scoping review to identify quality indicators used in national healthcare quality monitoring programs for the rural populations in countries around the world.
  ○ Prior to this review it was unknown whether there are rural specific quality indicators utilised for measuring the quality of care provided to older adults.
  ○ Identified quality indicators can assist countries such as Australia to consider adoption of relevant indicators to drive improvements in the access and quality of healthcare that older adults that live in rural areas receive.

## 1 Introduction

A large proportion of older adults globally live in rural and remote areas, with 70%–80% of populations in lower middle‐income countries such as Papua New Guinea, Vietnam, and Zambia [1] and 20%–30% of populations in Australia, Canada, and the United States of America (USA) [2, 3, 4]. While many prefer to age in place, older adults living in rural and remote areas face barriers common across the world due to limited access to high‐quality healthcare, primarily due to a lack of workforce and infrastructure [1, 5, 6]. These challenges to healthy ageing in rural and remote areas, such as fewer specialist medical services, allied health services, long‐term care homes, hospital facilities, and in‐home support services, are compounded by the need for long‐distance travel, making rural ageing more complex than in metropolitan areas [1, 7, 8, 9].

Governments worldwide aim to improve healthcare access in rural areas [1], recognising equity and accessibility as key quality dimensions best assessed by associated quality indicators (QIs) [10]. QIs are standardised, evidence‐based measures that reflect change or improvement in healthcare quality to facilitate monitoring and benchmarking of care quality [11, 12]. While national monitoring programs for older adults in long‐term care are increasingly considered, with currently 13 countries, including the USA and Australia [13, 14] using QI programs, it is unclear if QIs are being used to measure the care of older adults in rural and remote settings. Additionally, participation by rural hospitals and healthcare services is also limited by lower case numbers and workforce shortages to report on QIs [15]. As a result, QIs routinely used in rural hospitals and health services are not comparable to health services in metropolitan settings, where the need to establish rural‐specific QIs or a dedicated rural QI reporting program has been acknowledged [15, 16].

This scoping review aimed to answer the question, ‘Are there QIs routinely used at the population level (e.g., by countries or large care organisations) to monitor the quality of healthcare provided to older adults living in rural and remote communities?’ To answer the aim, this review (i) identified QIs in programs that are routinely used for monitoring the quality and safety of care for older adults living in rural and remote settings, (ii) synthesised QIs into types, dimensions, and domains of care, and (iii) identified any tools associated with included QIs which were reported as the standard data collection tool used to routinely collect QI data.

## 2 Methods

A scoping review following a protocol registered on Open Science Framework [17], conducted between November 10th 2023 and June 14th 2024 and reported according to the Preferred Reporting Items for Systematic reviews and Meta‐analyses extension for Scoping Reviews (PRISMA‐ScR) [18]. Due to the small number of QIs measuring the same concept, the use of the Appraisal of Indicators through Research and Evaluation (AIRE) instrument to evaluate indicators was not required to identify superior QIs as detailed in the protocol.

### 2.1 Eligibility Criteria

QIs and associated tools routinely used to monitor the quality and safety of rural or remote healthcare of older adults (aged ≥ 65 years or ≥ 50 years old for Indigenous Populations), published in English were included. As definitions for rural and remote areas vary across countries (e.g., by population‐based density or distance‐based), we did not apply a standard definition but utilized the terms rural and remote for all countries as they are used to describe those areas of limited geographical accessibility.

QIs not specific to the care of older adults in rural or remote settings were excluded. For example, indicators measuring the care of older adults in all long‐term care homes, including metropolitan, rural, and remote areas (i.e., Australia's National Aged Care Mandatory Quality Indicator Program [13]), or generally measuring population healthcare in hospital or primary care settings were outside the scope of this review. QIs not routinely used to monitor rural or remote care of older adults were excluded. Routinely used was defined as being currently implemented within a healthcare system and repeatedly used (more than once) to monitor the quality and safety of care [19].

### 2.2 Search Strategy and Selection

A systematic search of Medline OVID Appendix 1 (Table A1), AgeLine (Table A2), and grey literature sources [17] was conducted. The search strategies were applied to databases on January 25th, 2024, where no study design or cohort age limit was applied. Records were limited to a publication date from 2012 onwards to ensure QIs identified were currently used. As it can take 4 years to develop QIs that appear to be valid and feasible according to experts, prior to additional years to consider the appropriateness of contextual implementation in countries and organisations, the search covered the last 12 years [20]. Records were uploaded to Covidence where two reviewers screened 10% of the records and met 80% agreement, prior to one reviewer screening the remaining records for inclusion [21].

Grey literature records were systematically collated according to a protocol [17], to enable two reviewers (JS and RF) to screen all grey literature records. The terms ‘rural’ and ‘remote’ were searched in the websites listed and for the first 10 pages of a Google search, the standard search term ‘rural care quality’ was used. Similarly, for lower middle‐income countries, a search of ‘(country name) care quality’ for each lower middle‐income country listed on the World Bank Group [22] to collate records from the first two pages of the search results for systematic screening. Two reviewers (JS and RF) used the standardised grey literature lists collated to screen and add any extra secondary links identified. Any discrepancies between the reviewers were discussed with involvement from a third reviewer (GC) as required.

### 2.3 Data Extraction

A standardized data extraction template was used to extract the country of origin, citation details, QI name, data collection methods (e.g., survey, administrative data, claims data), data availability, population, Donabedian type of indicator (i.e., structure, process, or outcome) [11] and Institute of Medicine (IOM) framework quality dimension (i.e., safe, efficient, patient‐centered, effective, efficient, timely, or equitable) [10, 11]. Donabedian type of indicator and the IOM framework were used as they are widely accepted frameworks for healthcare quality, which can be comparable to QI programs in other settings. Two reviewers extracted a 10% proportion of the included records to ensure 80% agreement prior to one reviewer completing the remaining data extraction with data checking from the second reviewer [21].

### 2.4 Data Synthesis

When QIs did not report the Donabedian type of indicator or their Institute of Medicine (IOM), two reviewers considered the indicator to categorise them as a structure, process, or outcome indicator according to Donabedian [11] and categorise them as primarily addressing safety, efficiency, patient‐centredness, effectiveness, or timely or equity in care according to the IOM framework [11]. QIs identified were then thematically analysed to determine the common domains (i.e., themes) of care that they were measuring and subdomains (i.e., subthemes). Characteristics of QIs were summarised while QI and associated data collection tools were descriptively synthesised according to their domain (i.e., accessibility, hospital readmission, resources, and service delivery) and subdomains if relevant (i.e., medications, community health service, emergency health service, patient experience, infection control, communication transfer, effective management, workforce, infrastructure, cost of service), and summarised in tables.

## 3 Results

**FIGURE 1:**
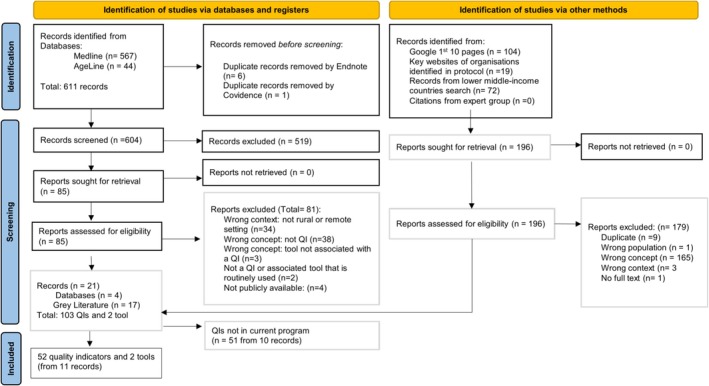
PRISMA flow diagram of study selection process [23].

Eleven unique records were eligible for inclusion, which captured nine QI programs in one of seven countries associated with 52 quality indicators and two tools (Figure 1). The unique records included were primarily grey sources (9 out of 11 records). Appendix 2 details key examples of records excluded at full text review and exclusion reasons.

Most QIs (42 of 52) identified were used in high‐income countries (i.e., USA *n* = 40 or Canada *n* = 2) with less (10 of 52) used in lower middle‐income countries (i.e., Papua New Guinea *n* = 1, Samoa *n* = 4, Timor‐Leste *n* = 1, Vietnam *n* = 1, and Zambia *n* = 3) (Table 1). The USA had three rural QI programs, namely, the Medicare Beneficiary Quality Improvement Program (MBQIP) managed by the Health Resources and Services Administration (HRSA) [24], Pennsylvania Rural Health Model (PARHM) [25] and Rural Emergency Hospital Quality Reporting Program (REHQRP) managed by The Centres for Medicare and Medicaid Services (CMS) [26], while other countries had one national program (Table 1).

**TABLE 1:** 52 Rural quality indicators and their attributes by country.

Country, program (total QIs)	QI name	Data collection method	Data availability	Population	Domain (subdomain)	IOM dimension	QI type
United States of America, HRSA Medicare Beneficiary Quality Improvement Program [24] (*n* = 30)	Median time from emergency department arrival to emergency department departure for discharged emergency department patients	Administrative data	Since 2010	Rural population	Access	Efficient	Process
	Patient left without being seen	Administrative data					
	Communication with nurses	Survey		Patients discharged from rural hospital	Service delivery (Patient Experience)	Person‐centred	Outcome
	Communication with doctors						
	Responsiveness of hospital Staff						
	Communications about medicines						
	Cleanliness of hospital environment						
	Quietness of hospital environment						
	Discharge information						
	Care transitions						
	Overall rating of hospital						
	Willingness to recommend						
	Influenza vaccination coverage amongst healthcare personnel (single rate for inpatient and outpatient settings)	Administrative data		Rural healthcare workers	Service delivery (Infection Control)	Safe	Outcome
	Commitment to antibiotic stewardship efforts	Patient Safety Component‐ Annual Hospital Survey		Rural hospitals			
	Antibiotic stewardship leaders						
	Priority antibiotic stewardship interventions						
	Antibiotic use policy or procedure						
	Pharmacy‐based interventions on antibiotic use						
	Engagement of bedside nurses in actions to optimise antibiotic use						
	Antibiotic use reports to prescribers						
	Antibiogram to prescribers						
	Information on antibiotic use, antibiotic resistance, and stewardship efforts is reported to hospital staff						
	Patient education on important side effects						
	Central line‐associated bloodstream infection (CLABSI) rate per 1000 central line days	Administrative data		Rural hospitals			
	CLABSI device utilisation ratio	Administrative data					
	Catheter‐associated urinary tract infection rate	Administrative data					
	CAUTI device utilisation ratio	Administrative data					
	Clostridioides difficile – laboratory identified events (intestinal infections)	Administrative data					
	Methicillin‐resistant *Staphylococcus Aureus* blood laboratory identified events (Bloodstream infections)	Administrative data					
	Emergency department transfer communication all or none composite calculation	Administrative data		Rural hospital emergency departments	Service delivery (Communication Transfer)	Timely	Process
United States of America, CMS Pennsylvania Rural Health Model [25] (*n* = 6)	Adults' access to preventive/ambulatory health services	Claims Data	2018–2024	Patients aged 20 years and older accessing rural services	Access	Effective	Process
	Follow‐up after emergency department visit for people with high‐risk multiple chronic conditions			Rural population	Access	Efficient	Process
	Pharmacotherapy for opioid use disorder			Patients 18 years and older accessing rural health services	Service Delivery (Effective Management)	Effective	Outcome
	Prevention quality indicator: chronic conditions composite						
	Plan all‐cause readmission				Hospital readmission	Safe	
	Hospital‐wide all‐cause unplanned readmission measure			Patients aged 65 years and older in rural hospitals	Hospital readmission	Safe	Outcome
United States of America, CMS Rural Emergency Hospital Quality Reporting Program [26] (*n* = 4)	Abdomen computed tomography use of contrast material	Administrative data	Since 2024	Rural population	Access	Efficient	Process
	Median time from emergency department arrival to emergency department departure for discharged emergency department patients	Administrative data & Claims Data					
	Facility 7‐day risk‐standardised hospital visit rate after outpatient colonoscopy			Patients aged 65 years and older in rural emergency hospitals	Hospital readmission	Safe	Outcome
	Hospital visits after hospital outpatient surgery						
Canada, Canadian Institute for Health Information [27] (*n* = 2)	Proportion of physicians in rural areas	Administrative data	1974–2022	Rural physicians	Resources (Workforce)	Equity	Structure
	Long term care facility location	Clinical Data	2022	Facilities in rural areas	Resources (Infrastructure)		
Papua New Guinea, National Department of Health [28] (*n* = 1)	Provincial health expenditure as a percentage of minimum cost of service delivery for rural health	Administrative data	2012–2015	Rural population	Resources (Cost of service)	Equity	Outcome
Samoa, Samoa Health System Strengthening Program [29] (*n* = 4)	Percentage of hypertensive patients, managed by rural health facilities, having their condition under control following WHO definition, disaggregated by gender.	Clinical data	2021–2024	Rural population	Service Delivery (Effective Management)	Effective	Outcome
	Percentage of patients in the hypertension and diabetes registry tracked and managed by rural health facilities following standardised disease management protocols, disaggregated by gender					Effective	Process
	Number of rural district hospitals with a multidisciplinary team in place			Rural hospitals and health facilities	Resources (Workforce)	Effective	Structure
	(Reduction of) stock‐outs (more than 2 weeks) of all essential drugs in rural health facilities	Administrative data			Access (Medications)	Safe	
Timor‐Leste, Ministry of Health (*n* = 1) [30]	Percentage of identified rural area with access to full packages of SISCa (Integrated Community Health Service)	Administrative data	Since 2009	Rural population	Access (Community Health Service)	Efficient	Structure
Vietnam, Ministry of Health [31] (*n* = 1)	Percentage of (rural) villages with active Village Health Worker	Administrative data	2011–2015				
Zambia, Ministry of Health [32] (*n* = 3)	Percentage of mobile outreaches conducted output	Administrative data	2016–2021	Rural population	Access (Emergency Health Service)	Timely	Process
	Percentage specialist outreaches conducted output						
	Percentage of rural health facilities with at least one qualified health worker			Rural workforce	Resources (Workforce)	Equity	Structure

Abbreviations: CAUTI, Catheter‐associated urinary tract infection; CLABSI, central line‐associated bloodstream infection; HRSA, Health Resources & Services Administration; QI, quality indicators; SISCa, integrated health service program in the community; WHO, World Health Organisation.

Three CMS QIs (i.e., two from REHQRP and one from PARHM) only measured data on older adults living in rural and remote areas (Table 2), whilst the other 49 QIs focused on the entire rural and remote population and were not specific to older adults. There were 47 QIs measuring in hospital care (including emergency, inpatient, and outpatient), and 5 QIs measuring available infrastructure, workforce, and healthcare expenditure for rural and remote populations. Most rural QIs (28 of 52) identified were ascertained using administrative or claims data, followed by survey (20 of 52) and clinical or medical record data (4 of 52) (Table 1). Most QIs measured outcomes (25 of 52), followed by process (19 of 52) and structure (8 of 52) concepts (Table 1). The most common IOM dimension of care health quality represented by QIs was safety (*n* = 22), followed by person‐centred (*n* = 10), effective (*n* = 7), efficient (*n* = 6), equity (*n* = 4), and timely (*n* = 3) care being least represented (Table 1). The 52 QIs represent four domains of care quality, namely, accessibility, hospital readmission, resources, and service delivery (Table 1).

**TABLE 2:** Rural quality indicators focusing on older adults.

Quality indicator name	Numerator	Denominator	Data required	Current program
Hospital Visits after Hospital Outpatient Surgery [33]	Unplanned hospital visits within 7 days after a surgery performed at a Rural Emergency Hospital that are: (1) an inpatient admission at a separate hospital that can admit patients; or (2) an emergency department visit, or observation stay at the REH or other hospital occurring after discharge. If more than one unplanned hospital visit occurs, only the first hospital visit within the outcome timeframe is counted in the outcome.	Eligible same‐day surgeries or cystoscopy procedures with intervention performed at REHs for Medicare FFS patients aged 65 years and older, except for eye surgeries and same‐day surgeries performed concurrently with high‐risk procedures.	Claims data	CMS Rural Emergency Hospital Quality Reporting Program
Facility 7‐Day Risk‐Standardised Hospital Visit Rate after Outpatient Colonoscopy [34]	All‐cause, unplanned hospital visits within 7 days of an outpatient colonoscopy performed at a Rural Emergency Hospital. We define a hospital visit as any emergency department visit, observation stay, or unplanned inpatient admission.	Outpatient colonoscopies performed at Rural Emergency Hospitals for Medicare patients aged 65 years and older.	Claims data	CMS Rural Emergency Hospital Quality Reporting Program
Hospital‐Wide All‐Cause Unplanned Readmission Measure [35]	30‐day readmission, defined as an inpatient readmission for any cause, except for certain planned readmissions, within 30 days from the date of discharge from an eligible index admission.	The measure includes admissions for Medicare beneficiaries who are 65 years and older and are discharged from all non‐federal, acute care inpatient US hospitals (including territories) with a complete claims' history for the 12 months prior to admission.	Claims data	CMS Pennsylvania Rural Health Model

Abbreviations: CMS, Centres for Medicare and Medicaid Services; REH, Rural Emergency Hospital.

Two standardised data collection tools were identified, both used to routinely collect QI data for a portion of QIs in the HRSA MBQIP. The first data collection tool was a survey that examined patients' experiences in hospital, where data for seven out of 10 person‐centred QIs were collected at hospital discharge [36]. The second data collection tool was The National Healthcare Safety Network, Patient Safety Component—Annual Hospital Survey, which was used to collect data for 10 infection control QIs as part of assessing antibiotic stewardship of small rural hospitals [36, 37]. From 103 QIs identified (Figure 1), 52 QIs were in current programs and 51 QIs were not in current programs; the common domains identified were accessibility, hospital admission, resources, and service delivery, and they were used for descriptive reporting on the 52 QIs in current programs.

### 3.1 Accessibility

There were 11 QIs measuring the accessibility of rural and remote healthcare. The USA had three rural CMS programs for rural hospitals with six QIs measuring the process of accessing emergency or preventative health services. Zambia measured the number of emergency mobile and specialist outreaches conducted (Table 1). Vietnam and Timor‐Leste measured the structural accessibility to health workers or a package of community health services. Samoa measured access to essential medications (Table 1).

### 3.2 Hospital Readmission

There were four QIs used by USA QI programs [25, 26] to measure rates of hospital readmission. Three QIs (two from the REHQRP and one from PARHM) measured either all unplanned hospital visits from older adults (i.e., aged 65 years and over) or unplanned hospital visits within 7 days of an outpatient colonoscopy or outpatient surgery amongst older adults (Tables 1 and 2). One QI from the PARHM broadly captured unplanned all‐cause readmissions to rural hospitals amongst the whole adult (aged 18 and over) rural population (Table 1).

### 3.3 Resources (Population, Workforce, Infrastructure, Cost of Care)

Five QIs measured the needs or availability of resources required to provide quality rural healthcare (Table 1). The QIs either measured the workforce in place, the cost of care, or the presence of long‐term care facilities. Canada measured the percentage of residents living in rural areas compared to the total population to assist with health system planning to improve access and quality of healthcare. Canada, Zambia, and Samoa had a workforce indicator. With regards to workforce measures, Canada's indicator focuses on the proportion of physicians in rural areas while Samoa and Zambia's indicators focus specifically on the workforce in facilities such as rural hospitals with multidisciplinary teams or rural facilities with at least one qualified health worker, respectively. Papua New Guinea measured the provincial health expenditure as a percentage of the cost of rural health service delivery. Canada measured the structural QI of long‐term care facilities in rural areas.

### 3.4 Service Delivery (Infection Control, Communication Transfer, Patient Experience, Effective Management)

All 32 QIs identified for measuring service delivery were part of the HRSA MBQIP [24] (Table 1). There was one process QI measuring accurate and timely communication transfer between rural emergency departments and the other healthcare facility to provide continuity of care and avoid errors and redundant tests. Four indicators measured effective management of conditions in specific populations with chronic conditions (*n* = 2 Samoa, *n* = 1 USA) or opioid use disorder (*n* = 1 USA). Ten patient experience QIs were collected by a patient's perspective of care survey tool and measured the communication by nurses and doctors, staff responsiveness, explanation of medicines, information provision on recovery at home and care after leaving the hospital, cleanliness and quietness of the hospital, overall rating of experience, and willingness to recommend the hospital [36]. Seventeen indicators measured the infection control processes for influenzas through staff vaccination, antibiotic use, as well as occurrence of hospital‐acquired infections (Table 1). A total of 10 out of 17 QIs were collected via a survey on antibiotic use (Table 1).

## 4 Discussion

This comprehensive scoping review identified three rural‐specific QIs that have been developed and used in America to measure safe hospital outcomes for older adults, namely hospital readmissions as a proportion of older adults with unplanned readmissions to the hospital or early readmission (within 7 days) after outpatient surgery or colonoscopy. Given that older adults account for a significant proportion of the rural and remote populations and are a vulnerable population group, the identified rural‐specific QIs could assist other countries such as Australia in monitoring the quality of care provided to older adults in rural settings [1, 2, 3, 4]. These measures capture the identification and avoidance of complications following certain procedures and potentially poor transitions back to home, which can be flags for high mortality risk, loss of functional independence, and healthcare costs [38, 39]. Using these measures adapted to their countries can enable rural hospitals to participate in monitoring programs and provide data‐driven evidence for improving resourcing if benchmarks are not met [15, 16].

Additionally, as frameworks to strengthen rural health systems in Australia [40] and globally [41] have identified the need for adequate accessibility, resources, and service delivery, these aspects could be measured by the rural‐specific QIs identified. Particularly, the accessibility of essential medicines and laboratory investigations, which are measured in Samoa, are basic health needs for the management of health conditions that should be considered in other countries to capture the resources required. As ageing and chronic disease prevalence rises, this impacts the demand for specialist care, in‐home care, and long‐term care [42], and it would also be prudent to examine accessibility and service delivery provided amongst these services for older adults through rural QI programs. Canada has a QI measuring long‐term care facilities close to home, reported independent of geographical location, despite having province/territory data which could also be considered by other countries.

Accessibility indicators specifically addressing how rural living older adults access healthcare also need to be considered and measured and may need to be considered based on the applicability to the country. For example, measuring mobile and specialist outreaches like Zambia may also be relevant for other countries that provide these services to their rural communities and can also overcome inequalities in workforce distribution. An example of this is rural and remote Australian communities that do not have access to health services within a 60 min drive with a motor vehicle and rely upon the royal flying doctors service [43]. To ensure this crucial transportation service continues in its capacity to serve the ageing rural population, the example Australian QI could be a transport QI used to measure the efficiency of this service in rural QI programs in Australia.

As resourcing rural healthcare is important globally [44], particularly after COVID‐19 [45], many countries could consider the proportion of health expenditure spent on rural health care, which is a QI currently only measured in Papua New Guinea based on its provinces. These QIs identified utilized administrative and claims‐based data, which reduces the burden on staff in gathering data, suggesting that they could be feasible in assisting rural and remote communities in participating in rural QI monitoring programs to facilitate improvement of the care of older adults living in these communities.

Most QIs were part of American QI programs focused on the processes and outcomes of hospital healthcare (Table 1). These programs aim to assist rural hospitals in achieving appropriate resources and funding allocation by measuring and reporting on basic outcome measures of safe hospital healthcare [46]. Therefore, hospital QIs were overrepresented in the findings, with a lack of quality QIs that measure other aspects of healthcare such as rural home care services and residential aged care services. The consideration of structural indicators from other countries such as Canada and the lower middle‐income countries with relevant QIs as mentioned above could assist the three American QI programs in also measuring structural aspects such as the resources and workforce required to provide the processes and outcomes measured.

### 4.1 Strengths and Limitations

This is the first systematic scoping review of QIs from rural programs focused on measuring the quality of healthcare provided to older adults living in rural and remote communities.

Whilst information published in languages other than English or in internal systems not accessible to the public may have limited the representation of QIs, particularly from lower middle‐income countries in this scoping review, both electronic databases and an extensive list of grey literature sources were used and returned a large number of results. Given that the aim of this review was to identify programs dedicated to monitoring rural healthcare quality and not necessarily programs that monitor healthcare in general, it is possible that other QIs that are used broadly across both rural and metropolitan settings may not be captured by this review. Nevertheless, QIs across four broad domains important to monitoring the care for older adults were identified.

### 4.2 Implications for Practice

Countries that have resident minimum data sets, capturing care of older adults residing in long‐term care, should consider whether it is possible to utilise claims data to report on the three rural quality indicators using the numerator and denominator (Table 2) for monitoring hospital readmissions for older adults living in rural communities. For example, in Australia, the National Mandatory Aged Care Reporting Program reports on hospital readmissions as the number of emergency department presentations or hospital admissions during the reporting quarter [47] rather than specifically reporting on unplanned readmission within 7 or 30 days (Table 2). Consistent centralised reporting could facilitate internal benchmarking that could identify sites which require additional resources and support whilst reducing the burden of individual sites to report to each funding program to access resources [48].

### 4.3 Implications for Research

As the USA is the only country that utilises the three‐hospital readmission rural specific QIs for older adults (Table 2), other countries need to consider the feasibility of collecting, reporting, and monitoring these QIs in Delphi and pilot studies. Additionally, the feasibility of QIs that capture the accessibility and timeliness of care, resources available, and service delivery of healthcare demands of older adults in rural communities, such as transportation, chronic disease management, specialist care, home care, and residential aged care, identified could be refined or developed in Delphi studies.

## 5 Conclusion

QIs used to monitor the quality of rural healthcare focused on accessibility, resources, service delivery, and hospital readmission. Indicators specific to older populations measuring early hospital readmission are suggested for worldwide consideration within country‐based contexts in future pilot studies. While the development of other QIs to capture the healthcare needs of rural‐living older adults will take considerable time, the application of the identified QIs to monitor care quality could potentially drive equitable investments in rural aged care.

## Author Contributions

J.S., M.C.I., G.E.C., and M.C. conceptualised the research question. J.S. and G.C. developed the search strategy. J.S. and R.L.F. screened records and extracted data. J.S. synthesised the findings and drafted the manuscript. All authors (J.S., R.L.F., M.C.I., M.C., X.L., G.E.C.) considered and interpreted the findings, revised the manuscript, and approved the final version for submission.

## Conflicts of Interest

The authors declare no conflicts of interest.

## Data Availability

Data sharing not applicable to this article as no datasets were generated or analysed during the current study.

## Appendix 1 Search Strategies

**TABLE A1:** Medline search strategies.

Lines numbers	Search strategy
1	Rural Health Services/or rural.mp. or Hospitals, Rural/or Rural Population/
2	“Medically underserved area”.mp. or Medically Underserved Area/
3	Non‐metropolitan.mp.
4	Remote Consultation/or remote.mp.
5	Rural Health/
6	1 or 2 or 3 or 4 or 5
7	Quality Indicators, Health Care/or “quality indicator”.mp.
8	Quality Assurance, Health Care/or “quality measure”.mp.
9	Clinical indicator*.mp.
10	7 or 8 or 9
11	6 and 10
12	Limit 11 to english language
13	Limit 12 to yr. = “2012 ‐Current”

**TABLE A2:** AgeLine search strategies.

Search numbers	Query	Limiters/Expanders	Last Run Via
S13	S6 AND S12	Expanders—Apply equivalent subjects Search modes—Boolean/Phrase	Interface—EBSCOhost Research Databases Search Screen—Advanced Search Database—AgeLine
S12	S7 OR S8 OR S9 OR S10 OR S11	Expanders—Apply equivalent subjects Search modes—Boolean/Phrase	Interface—EBSCOhost Research Databases Search Screen—Advanced Search Database—AgeLine
S11	TX quality assurance in healthcare	Expanders—Apply equivalent subjects Search modes—Boolean/Phrase	Interface—EBSCOhost Research Databases Search Screen—Advanced Search Database—AgeLine
S10	Quality benchmarks in healthcare	Expanders—Apply equivalent subjects Search modes—Boolean/Phrase	Interface—EBSCOhost Research Databases Search Screen—Advanced Search Database—AgeLine
S9	TX clinical indicator	Expanders—Apply equivalent subjects Search modes—Boolean/Phrase	Interface—EBSCOhost Research Databases Search Screen—Advanced Search Database—AgeLine
S8	TX quality measure	Expanders—Apply equivalent subjects Search modes—Boolean/Phrase	Interface—EBSCOhost Research Databases Search Screen—Advanced Search Database—AgeLine
S7	TX quality indicator	Expanders—Apply equivalent subjects Search modes—Boolean/Phrase	Interface—EBSCOhost Research Databases Search Screen—Advanced Search Database—AgeLine
S6	S1 OR S2 OR S3 OR S4 OR S5	Expanders—Apply equivalent subjects Search modes—Boolean/Phrase	Interface—EBSCOhost Research Databases Search Screen—Advanced Search Database—AgeLine
S5	TX rural healthcare or rural communities or rural areas	Expanders—Apply equivalent subjects Search modes—Boolean/Phrase	Interface—EBSCOhost Research Databases Search Screen—Advanced Search Database—AgeLine
S4	TX rural population or rural areas or rural communities	Expanders—Apply equivalent subjects Search modes—Boolean/Phrase	Interface—EBSCOhost Research Databases Search Screen—Advanced Search Database—AgeLine
S3	TX non‐metropolitan	Expanders—Apply equivalent subjects Search modes—Boolean/Phrase	Interface—EBSCOhost Research Databases Search Screen—Advanced Search Database—AgeLine
S2	TX medically underserved areas	Expanders—Apply equivalent subjects Search modes—Boolean/Phrase	Interface—EBSCOhost Research Databases Search Screen—Advanced Search Database—AgeLine
S1	TX rural OR TX remote	Expanders—Apply equivalent subjects Search modes—Boolean/Phrase	Interface—EBSCOhost Research Databases Search Screen—Advanced Search Database—AgeLine

## Appendix 2 Examples of Records Excluded at Full Text

**Table:**

Exclusion reason	Quality indicator name	Source
Wrong concept: Disaggregated data	Percentage of older people aged 60 or over living in rural and urban areas	World Health Organisation, 2024, The Global Health Observatory, Percentage of older people aged 60 or over living in rural and urban areas. Accessed on 11.04.2024 from https://www.who.int/data/gho/indicator‐metadata‐registry/imr‐details/percentage‐of‐older‐people‐aged‐60‐or‐over‐living‐in‐rural‐and‐urban‐areas
Wrong concept: No longer endorsed by National Quality Forum as a valid and reliable measure	Preventive Care and Screening: Screening for Clinical Depression and Follow‐Up Plan Preventive Care and Screening: Body Mass Index (BMI) Screening and Follow‐Up	National Quality Forum 2022 Key Rural Measures: An Update List of Measures to Advance Rural Health Priorities, Draft 1, page 1–26
Wrong concept: No longer part of a rural program	Median Time to Transfer to another Facility for Acute Coronary Intervention	Stratis Health. MBQIP Measures Fact Sheets 2016. Accessed March 15th 2024https://worh.org/wp‐content/uploads/2016/06/MBQIP‐Measures‐Fact‐Sheets‐OP3.pdf
Quality indicator information not publicly available in English	Doctors for rural populations ‐minimum Doctors for rural populations—maximum Count of rural service doctors Rural service doctors for rural population	Romero‐Alvarez D, López‐Cevallos DF, Torres I. Doctors for the people? The problematic distribution of rural service doctors in Ecuador. Health Policy Plan. 2023 Aug 2;38 (7):851–861. doi: https://doi.org/10.1093/heapol/czad040. PMID: 37402618

## References

[1] World Health Organisation , “Percentage of Older People Aged 60 or Over Living in Rural and Urban Areas. World Health Organisation Data Platform”, https://platform.who.int/data/maternal‐newborn‐child‐adolescent‐ageing/indicator‐explorer‐new/MCA/percentage‐of‐older‐people‐aged‐60‐or‐over‐living‐in‐rural‐and‐urban‐areas.

[2] AIHW , “Older Australians, 2024”, https://www.aihw.gov.au/reports/older‐people/older‐australians.

[3] A. Symens Smith and E. Trevelyan , “In Some States, More Than Half of Older Residents Live in Rural Areas,”.

[4] Statistics Canada , “In the Midst of High Job Vacancies and Historically Low Unemployment, Canada Faces Record Retirements From an Aging Labour Force: Number of Seniors Aged 65 and Older Grows Six Times Faster Than Children 0–14”, https://www150.statcan.gc.ca/n1/daily‐quotidien/220427/dq220427a‐eng.htm.

[5] M. Lahr and C. Henning‐Smith , “Barriers to Aging in Place in Rural Communities: Perspectives From State Offices of Rural Health, 2021”.

[6] OECD/European Union , “Chronic Diseases and Disabilities Among Older People,” in Health at a Glance: Europe 2022: State of Health in the EU Cycle (OECD Publishing, 2022).

[7] S. Krasniuk and A. M. Crizzle , “Impact of Health and Transportation on Accessing Healthcare in Older Adults Living in Rural Regions,” Transportation Research Interdisciplinary Perspectives 21 (2023): 100882, 10.1016/j.trip.2023.100882.

[8] I. Blackberry and N. Morris , “The Impact of Population Ageing on Rural Aged Care Needs in Australia: Identifying Projected Gaps in Service Provision by 2032,” Geriatrics (Basel) 27 8, no. 3 (2023): 47, 10.3390/geriatrics8030047. 37218827 PMC10204523

[9] National Rural Health Alliance , “Rural Health in Australia Snapshot, 2023”.

[10] Institute of Medicine Committee on Quality of Health Care in America , Crossing the Quality Chasm: A New Health System for the 21st Century (National Academies Press, 2001). 25057539

[11] Agency for Healthcare Research and Quality , “AHRQuality Indicators: Measures. U.S. Department of Health and Human Services”, https://qualityindicators.ahrq.gov/measures/qi_resources#:~:text=Quality%20Indicators%20(QIs)%20are%20standardized,track%20clinical%20performance%20and%20outcomes.

[12] Helsedirektoratet , “National Healthcare Quality Indicators: A Statistical Approach Towards Better Quality”, https://www.helsedirektoratet.no/english/national‐health‐care‐quality‐indicators.

[13] Department of Health and Aged Care , “National Aged Care Mandatory Quality Indicator Program (QI Program). Australian Government”, https://www.health.gov.au/our‐work/qi‐program.

[14] Centers for Medicare & Medicaid Services , “Skilled Nursing Facility (SNF) Quality Reporting Program (QRP) Measures and Technical Information. United States Government”, https://www.cms.gov/medicare/quality/snf‐quality‐reporting‐program/measures‐and‐technical‐information.

[15] National Quality Forum , “Key Rural Measures: An Updated List of Measures to Advance Rural Health Priorities, 2022”.

[16] M. Baernholdt , B. M. Jennings , E. Merwin , and D. Thornlow , “What Does Quality Care Mean to Nurses in Rural Hospitals?,” Journal of Advanced Nursing 66, no. 6 (2010): 1346–1355, 10.1111/j.1365-2648.2010.05290.x. 20546364 PMC3624755

[17] J. Suen , R. Fernando , M. C. Inacio , G. E. Caughey , and M. Crotty , Identification of Quality Indicators Used to Monitor, Evaluate and Improve the Quality of Rural and Remote Care for Older People: A Scoping Review Protocol, (Open Sciences Framework, 2024), 10.17605/OSF.IO/6RTCD.41214894

[18] A. C. Tricco , E. Lillie , W. Zarin , et al., “PRISMA Extension for Scoping Reviews (PRISMA‐ScR): Checklist and Explanation,” Annals of Internal Medicine 169, no. 7 (2018): 467–473, 10.7326/M18-0850. 30178033

[19] T. J. H. Lathlean , M. C. Inacio , J. Westbrook , et al., “Quality Indicators to Monitor the Quality and Safety of Care for Older People: A Scoping Review Protocol,” JBI Evidence Synthesis 22, no. 9 (2024): 1857–1865, 10.11124/jbies-23-00212. 38832459

[20] M. N. Marshall , P. G. Shekelle , E. A. McGlynn , S. Campbell , R. H. Brook , and M. O. Roland , “Can Health Care Quality Indicators Be Transferred Between Countries?,” Quality & Safety in Health Care 12, no. 1 (2003): 8–12, 10.1136/qhc.12.1.8. 12571338 PMC1743668

[21] B. J. Shea , B. C. Reeves , G. Wells , et al., “AMSTAR 2: A Critical Appraisal Tool for Systematic Reviews That Include Randomised or Non‐Randomised Studies of Healthcare Interventions, or Both,” BMJ 358 (2017): j4008, 10.1136/bmj.j4008. 28935701 PMC5833365

[22] The World Bank , “World Bank Country and Lending Groups. The World Bank Group”, https://datahelpdesk.worldbank.org/knowledgebase/articles/906519‐world‐bank‐country‐and‐lending‐groups.

[23] M. J. Page , J. E. McKenzie , P. M. Bossuyt , et al., “The PRISMA 2020 Statement: An Updated Guideline for Reporting Systematic Reviews,” BMJ 372 (2021): n71, 10.1136/bmj.n71. 33782057 PMC8005924

[24] Health Resources & Services Administration , “Medicare Beneficiary Quality Improvement Project”, https://www.hrsa.gov/rural‐health/grants/rural‐hospitals/medicare‐beneficiary‐quality‐improvement.

[25] Centers for Medicare & Medicaid Services , “Pennsylvania Rural Health Model,” https://www.cms.gov/priorities/innovation/innovation‐models/pa‐rural‐health‐model.

[26] American Medical Association , “Rural Emergency Hospital Quality Program Specifications Manual,” (2024) Release Notes Version 2.0.

[27] Canadian Institute of Health Information , “Indicator Library”, https://www.cihi.ca/en/access‐data‐and‐reports/indicator‐library?keyword=rural&type_of_care=All&place_of_care=All&population_group=All&health_conditions_outcomes=All&health_care_quality=All&health_system_overview=All&hsp_framework=All&acronyms_databases=All&sort_by=title&items_per_page=20.

[28] Government of Papua New Guinea , “National Health Plan (2011–2020) 2019 Sector Performance Annual Review, 2020”.

[29] World Bank Group , “Samoa Health System Strengthening Program”, https://projects.worldbank.org/en/projects‐operations/project‐detail/P164382.

[30] TALITI , “National Health Sector Strategic Plan 2011–2030, 2011”.

[31] “Plan: For People's Health Protection, Care and Promotion 2016–2020, 2016”.

[32] Zambia Ministry of Health , “Zambia National Health Strategic Plan 2017–2021”.

[33] Centers for Medicare & Medicaid Services Measures Inventory Tool , “Hospital Visits After Hospital Outpatient Surgery”, https://cmit.cms.gov/cmit/#/MeasureView?variantId=12917&sectionNumber=1.

[34] Centers for Medicare & Medicaid Services Measures Inventory Tool , “Facility 7‐Day Risk‐Standardized Hospital Visit Rate After Outpatient Colonoscopy”, https://cmit.cms.gov/cmit/#/MeasureView?variantId=12916&sectionNumber=1.

[35] Centers for Medicare & Medicaid Services Measures Inventory Tool , “Hospital‐Wide All‐Cause Unplanned Readmission Measure (HWR)”.

[36] Centers for Medicare & Medicaid Services , “HCAHPS: Patients' Perspectives of Care Survey”, https://www.cms.gov/medicare/quality/initiatives/hospital‐quality‐initiative/hcahps‐patients‐perspectives‐care‐survey.

[37] National Healthcare Satefy Network , “Patient Safety Component—Annual Hospital Survey”, https://www.cdc.gov/nhsn/forms/57.103_pshospsurv_blank.pdf.

[38] A. Bortolani , F. Fantin , A. Giani , et al., “Predictors of Hospital Readmission Rate in Geriatric Patients,” Aging Clinical and Experimental Research 36, no. 1 (2024): 22, 10.1007/s40520-023-02664-9. 38321332 PMC10847193

[39] F. Cilla , I. Sabione , and P. D'Amelio , “Risk Factors for Early Hospital Readmission in Geriatric Patients: A Systematic Review,” International Journal of Environmental Research and Public Health 20, no. 3 (2023): 1674. 36767038 10.3390/ijerph20031674 PMC9914102

[40] Standing Council on Health , “The National Strategic Framework for Rural and Remote Health, 2011”.

[41] D. R. Pamungkas , B. O'Sullivan , M. McGrail , and B. Chater , “Tools, Frameworks and Resources to Guide Global Action on Strengthening Rural Health Systems: A Mapping Review,” Health Research Policy and Systems 21, no. 1 (2023): 129, 10.1186/s12961-023-01078-3. 38049824 PMC10694960

[42] D. Asante , C. S. McLachlan , D. Pickles , and V. Isaac , “Understanding Unmet Care Needs of Rural Older Adults With Chronic Health Conditions: A Qualitative Study,” International Journal of Environmental Research and Public Health 20, no. 4 (2023): 3298, 10.3390/ijerph20043298. 36833993 PMC9960497

[43] F. W. Gardiner , A. M. Richardson , L. Bishop , et al., “Health Care for Older People in Rural and Remote Australia: Challenges for Service Provision,” Medical Journal of Australia 211, no. 8 (2019): 363–364, 10.5694/mja2.50277. 31318054

[44] I. Couper , M. I. Lediga , N. B. Takalani , et al., “Shaping the Future Rural Healthcare Landscape: Perspectives of Young Healthcare Professionals,” Rural and Remote Health 24 (2024): 8792, 10.22605/RRH8792. 39871548

[45] J. E. Fried , D. T. Liebers , and E. T. Roberts , “Sustaining Rural Hospitals After COVID‐19: The Case for Global Budgets,” Journal of the American Medical Association 324, no. 2 (2020): 137–138, 10.1001/jama.2020.9744. 32520306 PMC7483972

[46] Rural Health Information Hub , “Rural Healthcare Quality, 2024”, https://www.ruralhealthinfo.org/topics/health‐care‐quality.

[47] The Australian Institute of Health and Welfare , “Residential Aged Care Quality Indicators—Annual Report 2023–24, 2024”.

[48] K. Woolcock , J. Gregg , and A. Groth , “Policy Alignment for Place‐Based Solutions for Better Health Outcomes in Rural and Remote Communities, 2025”.

